# The Effects of Emotional Design on Multimedia Learning and Appreciation of Chinese Poetry

**DOI:** 10.3389/fpsyg.2021.621969

**Published:** 2021-08-05

**Authors:** Yi Wang, Zhijin Zhou, Shaoying Gong, Dandan Jia, Jing Lei

**Affiliations:** ^1^Key Laboratory of Adolescent Cyberpsychology and Behavior (Central China Normal University), Ministry of Education, Wuhan, China; ^2^School of Psychology, Central China Normal University, Wuhan, China; ^3^Dengzhou No. 1 Middle School, Dengzhou, China

**Keywords:** emotional design, emotion induction, appreciation of chinese poetry, positive emotions, cognitive affective theory of learning with media (CATLM)

## Abstract

Painting, music, literature, and other art forms embody the essence of human wisdom and induce esthetic experience, among which poetry is inherently creative, because it contains a wealth of symbols, imageries, insights, and so forth. The appreciation and learning of Chinese poetry is an important part of the curriculum in secondary schools. However, studies have mainly focused on textual characters of poetry, with little literature focusing on esthetic appreciation and in-depth learning of poetry. In this vein, we ask whether emotional designs will promote the appreciation and learning of Chinese poetry. To answer this question, we explored the influence of the combination of external emotion induction (positive and neutral movie clips) and internal colorful design (chromatic and achromatic) on esthetic preference and learning of poetry. One hundred and sixty-six participants (14–15 years old) were randomly assigned to one of four conditions created by two factors (external emotion induction and internal colorful design). The results showed that the combination of external emotion induction and internal colorful design promoted positive emotions, retention, and transfer performances of learners. Furthermore, perceived difficulty of learners decreased significantly when external emotional induction and internal colorful design were both positive. Consequently, these findings indicated that emotional designs in multimedia facilitated the learning performance of middle school students in Chinese poetry, and supported the cognitive-affective theory of learning with media. This research was a preliminary exploration of emotional design in humanities.

## Introduction

The crystallization of human wisdom, paintings, music, literature, and other art forms can induce esthetic experiences in people. Poetry, which contains abundant symbols, icons, and imageries, is a creative language and an indispensable form of literature. In the current literature, studies have focused more on the superficial textual characters of poetry, such as rhyme (Jacobs, 2015), and its influence on poetry appreciation. Rhyme of poems in different languages, such as Chinese (Chen et al., 2016; Chen and Yang, 2017), English (Xue et al., 2019), German (Obermeier et al., 2013; Lüdtke et al., 2014), and Spanish (Navarro-Colorado, 2018), has received extensive attention from researchers. However, less attention has been directed to the esthetic appreciation and in-depth learning of poetry in multimedia learning classrooms. To fill this gap, we investigated the impact of emotional designs in the multimedia environment on the appreciation and learning of Chinese poetry, which belongs to liberal education.

### The Rhythm of Chinese Poetry and Its Impacts on Appreciation

The current empirical research on Chinese poetry mainly focuses on the congruency of rhyme and its expectation effect, and indicates that the rhyming effect works throughout the reading process of Chinese poetry. Specifically, top-down expectations about the rhyme scheme modulated early phonological coding of Chinese characters and semantic access thereafter (Chen et al., 2016; Chen and Yang, 2017). In addition, the perception of rhyme does not seem to be influenced by relevant experiences: poetry is the spoken form of music songs. Although subjects who have been trained in music for years can process rhyme in Chinese poetry more quickly (Zhang et al., 2020), pupils in primary schools have acquired rhythm information on a Chinese poem (e.g., coherent repetition, stress pattern) implicitly (Li et al., 2009).

Moreover, rhyming and regular metered poetry can promote the esthetic appreciation of Chinese poetry (Gao and Guo, 2018). Gao and Guo (2018) employed *QiJue*, which is characterized by strict rhyme schemes and constitutes a well-structured prosodic hierarchy in accordance with the features of ancient Chinese poetry, and examined the mechanism of appreciation of beauty of Chinese poetry. They found that, compared with reading prose, appreciating *QiJue* promoted the activation of the bilateral insula and the left inferior orbitofrontal cortex, which plays a significant role in the neural basis of esthetic appreciation.

### Impacts of Other Factors on Poetry Appreciation

Rhythm may only be one of the influential factors affecting the esthetic appreciation of poetry. Besides rhythm, other features in poetry, such as e key emotional tonality and motifs (or themes), can impact esthetics as well. To illustrate, Kraxenberger and Menninghaus (2017) asked adult participants to respond to two self-reported items (“How beautifully is the poem written?” and “How much do you like this poem?”) to evaluate the esthetic appreciation of poems with different emotional tonalities. They found that, compared with happy poems, the subjects had higher esthetic appreciation toward sad poems. Regarding the influence of motifs on poetic esthetic appreciation, Lüdtke et al. (2014) investigated the esthetic appreciation of four different themes (morning, space, stillness, and city). Three self-reported items were completed by readers to evaluate poems from three different aspects: beauty, affection (liking), and attractiveness (wanting). The results indicated that the stillness-themed poems [Tieck's “Im Windsgeräusch, in stiller Nacht,” 1796 (The Sound of Wind in the Silent Night)] evoked significantly higher esthetic appreciation than other themes.

In sum, the current literature mainly examines influences of surface features of poetry, such as rhythm, emotional tonality, and motif, on the appreciation of poetry. However, few studies have focused on the deep comprehension of poetry texts and their transfer applications (Xue et al., 2020), especially in the study of Chinese poetry. For example, existing studies focus more on whether there is a violation of the rhyme on the level of phonology (Chen et al., 2016), and whether they are esthetically beautiful merely by subjective evaluation (Gao and Guo, 2018). Less focus is placed on semantics, such as meanings of poetry beyond its literary meaning [Seyed-Gohrab (2012) in certain cultural context]. This may be attributed to the following reasons: the language of poetry, by its very nature, tends to compress, inclining toward the condensation of metaphorical language without syntax or connectives, resulting in subjective and open-ended poetry comprehension and appreciation (Peskin, 1998). Thus, the obscurity of language, reflected by imageries, insights, and icons of poets, leads to greater demands on cognitive resources of readers, and the interpretation is tightly bound up to prior knowledge of poetry of readers (Peskin, 1998; Piirto, 2011). In addition, the role of culture in the comprehension of figurative language, which might be referred to a general relationship between the language and the body, can be another reason. For example, differences in Italian and Persian abstract languages were revealed when it comes to the comprehension of the embodied language (Ghandhari et al., 2020). There are similar examples in Chinese classical poetry, e.g., the action of “折柳,” with the literary meaning of “pick willow branches,” usually means to persuade one's friends to stay and expresses the feelings of missing, which may exist in context of Chinese culture exclusively. Therefore, to enhance the deep comprehension of learners of poetry, we presented the imageries and scenes depicted in a rhyming and regular metered, idyllic Chinese poem in a Chinese culture context (a middle school in Chinese mainland). A Chinese poem was presented to learners visually and intuitively via an instructional flash animation in a multimedia environment to complement the compressed language, reducing demands on readers' cognitive resources, the obscurity of language, and fostering an in-depth understanding of the poem.

### Theoretical Framework

Multimedia technologies are increasingly used in educational settings. Furthermore, emotional designs, instructional designs that use different design elements (e.g., pleasant colors and anthropomorphisms) to influence emotions of learners in the process of learning to improve academic performance of learners (Plass and Kaplan, 2016), have been widely employed across natural science disciplines (Um et al., 2012; Plass et al., 2014; Uzun and Yildirim, 2018; Shangguan et al., 2020a).

Regarding the influence of emotions on learning performance, the emotions-as-facilitator-of-learning hypothesis (Um et al., 2012; Park et al., 2015; Knörzer et al., 2016) assumes that cognitive and learning processes, such as information processing, category sorting tasks, and creative problem-solving (Erez and Isen, 2002), can be enhanced *via* positive emotions. An influential extended framework, the cognitive-affective theory of learning with media (CATLM) (Moreno and Mayer, 2007) further proposes that affective and motivational factors are mediating factors between emotional designs and learning effects in multimedia learning. Therefore, emotion was integrated into the CATLM, and Moreno and Mayer (2007) put forward the emotional design hypothesis, which holds that in the process of learning, making basic elements visually attractive (e.g., pleasant colors or presenting them with shapes similar to human faces) within a learning material will initiate cognitive processing (Mayer and Estrella, 2014). That is, cognitive processing during learning can be improved through the emotional design of elements in the multimedia learning material; thus, improving the learning effect. Therefore, the emotional design hypothesis has been confirmed in research (Mayer and Estrella, 2014; Gao, 2016).

In addition to the effect of internal emotional designs on learning material, external emotion-inducing methods, such as reading emotional text (Um et al., 2012; Park et al., 2015), viewing pictures and videos (Plass et al., 2014; Gong et al., 2017, Exp. 1), and recalling emotional autobiographical memories (Knörzer et al., 2016), are also effective ways to induce emotions of learners in education and also impact learning processes (Brose et al., 2012; Beege et al., 2018). The measurement of external emotion induction generally takes place after emotion induction and before multimedia learning. Although external emotion induction cannot run through the whole learning process continuously, researchers have consistently confirmed that external emotion induction can successfully induce positive emotions in learners (Um et al., 2012; Plass et al., 2014; Park et al., 2015; Knörzer et al., 2016; Schneider et al., 2016; Gong et al., 2017). The CATLM successfully explains some research on positive emotional designs in multimedia learning. For instance, Plass et al. (2014) reported that positive emotions induced by internal emotional design enhanced intrinsic motivation and comprehension performance. In addition, Um et al. (2012) found that positive emotions induced by external and internal emotional designs boosted intrinsic motivation and transfer performance of learners. However, some studies have suggested no differences in motivations (Kumar et al., 2016) or learning outcomes (Park et al., 2015; Kumar et al., 2016) between positive emotional design conditions and neutral ones, inconsistent with the proposals of the CATLM.

Meanwhile, the emotions-as-suppressor-of-learning hypothesis postulates that emotions may impair learning (Um et al., 2012). Consistent with this, cognitive load theory (Paas et al., 2003; Paas and Sweller, 2014; Kalyuga and Singh, 2016) predicts the opposite regarding learning outcomes where the positive emotions induced, compared with neutral emotions, may increase the extraneous cognitive load in learners (Rey, 2012), which is adverse to learning performance. Therefore, cognitive load theory is partially supported by previous studies (Schneider et al., 2018; Starkova et al., 2019).

The inconsistent findings in the literature may be due to the following reasons: first, the subjects of the learning material varied across studies. Most of existing research focuses on STEM (science, technology, engineering, and mathematics) subjects, such as the formation of lightning (Gong et al., 2017; Shangguan et al., 2020a), ATP structure and synthesis (Park et al., 2015; Knörzer et al., 2016; Stark et al., 2018), mechanics, and efficacy (Uzun and Yildirim, 2018), with limited focus on humanities. Second, learning in a multimedia environment involves a relatively complicated cognitive process. Therefore, in addition to induced emotions and motivation, other factors specific to the attributes of disciplines that affect learning outcomes may be present. For example, understanding and appreciation of readers of poetic texts are influenced by surface psycholinguistic features (Xue et al., 2020). Third, the internal emotional design elements of the learning material used vary in different studies. That is, some employed a combination of multiple design elements (Gong et al., 2017; Shangguan et al., 2020a), while others distinguished between different elements, such as anthropomorph or color (Heidig et al., 2015; Gong et al., 2017, Exp. 2). It has been proven that colors affect emotions, and that warm colors are deemed as stimulating and active (Kaya and Epps, 1998). Instructional designers also recruit warm colors to draw the attention of learners (Lohr, 2007). Color should be considered when manipulating emotional designs as posited by the ecological valence theory of human color preference (Palmer and Schloss, 2010), which believes that color contains useful information about an object that leads to approach-avoidance behavior of people. For example, people usually prefer red apples to green ones because the former may represent maturity and delicacy. In addition, approach-avoidance behavior is context-specific in which red in the orchard means maturity, while red signifies stopping on roads. Research on visual esthetics has also verified that people prefer color hue, saturation, brightness, and so forth, with people generally preferring harmonious color combinations (Palmer et al., 2013). Research has also confirmed the role of color in emotional design. To illustrate, Münchow et al. (2017) applied warm color designs to learning material on neuroanatomical topics and indicated that, compared with gray-scaled designs, warm color designs improved comprehension and transfer performance of learners. Plass et al. (2019) further verified that warm colors in teaching games were related to the positive emotions of participants. In addition, Wong and Adesope (2021) conducted a meta-analysis on emotional designs and found that pleasant colors were indeed an effective design principle.

Chinese poetry, which is a concentrated expression of Chinese art and culture, is loved by many literati and people at home and abroad. The terms used in Chinese poetry are appreciated esthetically. When writing poems, poets usually create connections between their inner feelings and objective external images in a metaphorical way, which is a creative process. The poet also experiences an insight, which involves restructuring the problem in a different way and enables him to experience an “Aha” moment. Poetry also provides “Aha” moments for its readers who are moved by the creative works of the poet whose insight provides insight to readers (Piirto, 2011). Only when the historical spiritual meanings of symbols, imageries, and icons in poetry are remotely associated with the esthetic creation of poets would readers understand the specific meanings of these symbols, imageries, and icons; and they would then resonate more with poets (Piirto, 2011). Accordingly, appreciation and learning of poetry could be a creative process in itself, which can boost creativity of readers (Osowiecka and Kolańczyk, 2018). That is, emotionally designed learning materials where colorful scenes in Chinese poetry are visually presented may help learners gain perception of the poetry intuitively by assisting learners in establishing remote associations between imageries in ancient Chinese poetry and objective matters, thus, achieving insight into personal creations of the poet and fostering esthetic appreciation and deeper learning of poetry.

### Problem Statement, Research Question, and Hypotheses

Existing studies on emotional designs are almost entirely concerned with natural sciences. Therefore, whether the general principles of emotional designs in STEM subjects are applicable to humanities is in question. That is, can the combination of internal colorful design of poetry learning materials and external emotion induction induce positive emotions of learners? Furthermore, what are the effects of this combination on appreciation and learning of poetry? These were the main concerns addressed in this study.

According to Leder and Nadal (2014), information processed in an esthetic episode involves perceptual, cognitive, and emotional components. That is, the appreciation of poetry could be broadly reflected in esthetic appreciation whether the poem is beautiful and fascinating, and whether one likes it. Lüdtke et al. (2014), which may include beauty (beauty) and preference ratings (wanting, liking) (Chatterjee and Vartanian, 2014; Leder and Nadal, 2014). Esthetic preference, which was separated from beauty and was a major achievement in post-Kantian esthetics, (Kraxenberger and Menninghaus, 2017), mainly follows preferences of individuals and is more closely related to emotional components. Neurasthenics confirmed that several brain regions associated with esthetic experience are implicated in emotion-valuation systems, such as the orbitofrontal and medial frontal cortices and insula (Blood and Zatorre, 2001; Chatterjee and Vartanian, 2014). Furthermore, affect could modulate the encoding and retrieval of information to form evaluations and judgments, especially when constructive processing is required (Forgas, 1995). When it comes to esthetic evaluations, artworks were more liked when they were preceded by positive primes compared with negative ones (Flexas et al., 2013), possibly because of a valence-congruency effect that indicates the transfer of affective reaction from primes to targets (Payne et al., 2005; Boukarras et al., 2020). Thus, we were curious whether emotionally designed poetry learning materials would foster the following esthetic preference of poetry.

In sum, this study aimed to investigate the influence of emotional designs on emotions of learners and esthetic preferences as well as the cognitive and learning outcomes when learning Chinese poetry in a multimedia environment with the CATLM. Specifically, the impact of the combination of external emotion induction (emotional films) and internal colorful design on positive emotions, esthetic preferences, and learning performances of learners of Chinese poetry was investigated.

Based on findings from previous studies, the following hypotheses were postulated: in the preliminary experiment, compared with internal neutral design (achromatic), internal colorful design would induce more positive emotions (H1) and esthetic preference (H2), and the positive film would evoke more positive emotions than neutral one (H3). In the following formal experiment, the combination of internal colorful design and external positive emotion induction could induce more positive emotions (H4) and esthetic preference (H5) in learners, leading to higher learning motivation, lower perceived difficulty (H6), and better learning performance (higher retention and transfer scores) (H7) compared with neutral conditions.

## Preliminary Experiment

A preliminary experiment was conducted to verify the emotional charges of the experimental materials. Specifically, the preliminary experiment was employed to determine whether the internal colorful design condition would induce more positive emotions and higher esthetic preference of the poem than those in the neutral condition, and whether the positive film evoked more positive emotions in learners than the neutral film.

### Verification of Learning Materials

#### Participants and Design

G*Power analysis (G*Power 3.1.9.2) was conducted to estimate the sample size (Faul et al., 2007) with an effect size (Cohen's *d*) of 0.8 and power of 0.9, as described by Cohen (1988). We aimed for a sample size of a minimum of 36 subjects in each condition. Eighty students (42 females, age: *M* = 14.91, *SD* = 0.86) with normal or corrected-to-normal vision in a middle school in Henan Province participated in the preliminary experiment. They were randomly assigned to two conditions: internal colorful design (*n* = 42) or internal neutral design (*n* = 38). Written informed consent was obtained from both the school principal and the parents before the experiment. The research protocol was approved by the Ethical Committee of the School of Psychology of Central China Normal University.

#### Selection and Design of Learning Materials

Six experienced Chinese teachers with an average teaching experience of 9 ± 2.67 years in a middle school rated the eight poems proposed by researchers on three aspects: difficulty, concreteness, and dynamics of poetic scenes, as well as emotions involved in the poem on a 7-point rating scale (rater reliability: Cronbach's α = 0.9). One poem that was not included in the existing syllabus and that had a difficulty level matching the syllabus at middle school level (*M* = 6.59 ± 0.32) was selected. It was a rhymed poem of the Tang Dynasty with highly concrete and dynamic scenes (*M* = 6.01 ± 0.52) and a relatively neutral emotional tone (*M* = 3.69 ± 0.72). The poem was Meng Haoran's 《夏日浮舟过陈大水亭》 (Boating to the Chen Pavilion on the Lake in Summer Evening). The relatively neutral poem content ensured that the perceived emotional involvement of the learning material was due to the manipulation of emotional design and not due to the poem itself (Beege et al., 2018).

**Figure 1:**
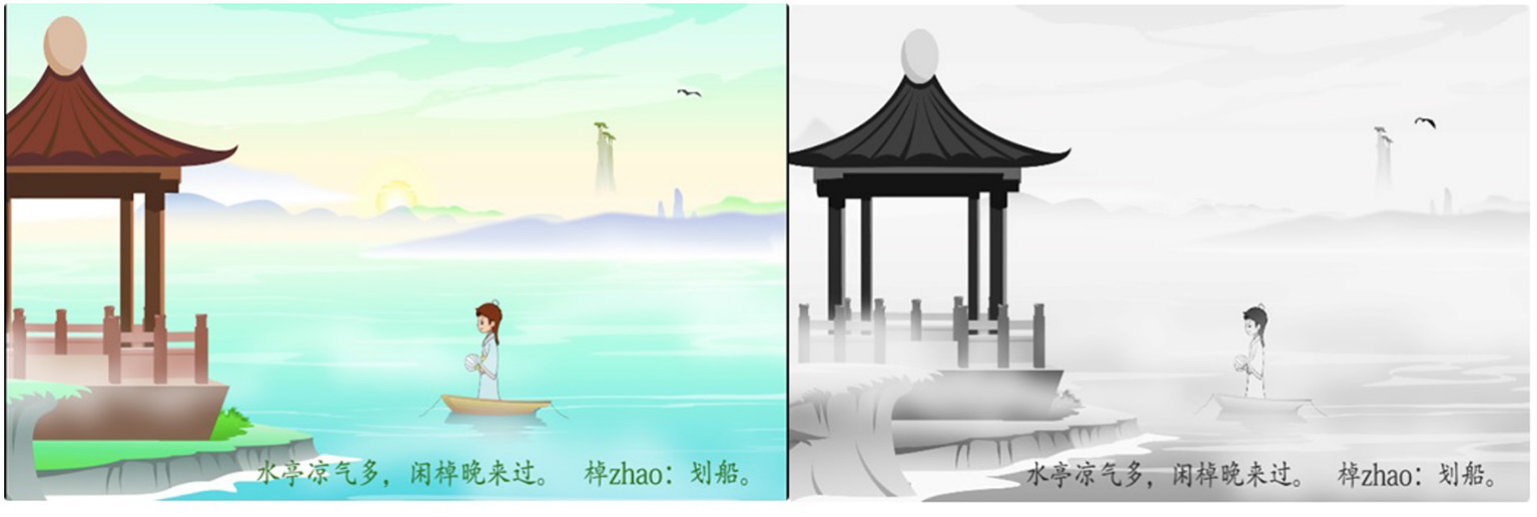
Screenshots of multimedia learning materials in the preliminary experiment: internal colorful design (IC) on the left and internal neutral design (IN) on the right.

The learning material of the poem was jointly compiled, according to the teaching syllabus and teaching experience, by two of the six teachers (Uzun and Yildirim, 2018). It contained 650 explanatory characters, of which 130 (including the title, author, phonetic annotation, and explanation of rare characters) were visually presented as captions. The material was presented in a flash animation with a length of 5 min and 16 s, and its production was completed under the guidance of teaching experts. It was designed in two versions, which were identical to each other, except for the colors. In the positive chromatic version, cold and warm colors were included (Um et al., 2012) according to the original colors of the objects; whereas, in the neutral version, the color was gray scale. Each version contained six dynamic pictures. Please see creenshots of the learning materials in Figure 1.

The learning material was presented at a fixed paced in three consecutive stages: first, the poem was recited with pronunciations and annotations of rarely used Chinese characters presented at the bottom of the corresponding pictures; second, the meanings of the poem were briefly explained; and finally, the overall thoughts and feelings in the poem were sublimated.

#### Emotional Measures

According to previous studies (Chaffar and Frasson, 2004; Gong et al., 2017), to check the manipulation of the mood induction, six items concerning positive emotions in a positive emotion self-report inventory (Gross and Levenson, 1995) were used (happy, excited, content, active, interested, and relaxed). The subjects were required to respond by indicating the extent to which they felt these six positive emotions in response to the learning material using a 9-point rating scale ranging from 1 (not at all) to 9 (very much). The positive emotion score was calculated by averaging the scores from the six responses above. This scale showed high internal consistency in the preliminary experiment (coefficient *a* = 0.89).

#### Esthetic Preference

The esthetic preference of the poem included two 7-point rating items (1 = strongly disagree, 7 = strongly agree) (Lüdtke et al., 2014; Kraxenberger and Menninghaus, 2017): “I like the poem” and “The poem is fascinating (attractive) to me.” The final score was calculated by averaging the scores of the two items. The internal consistency coefficients of the two items were 0.9.

#### Control Measure: Prior Knowledge of Chinese Poetry

The prior knowledge questionnaire on Chinese poetry was compiled by Chinese teaching experts in a middle school based on literature by Xu (2020) and Bao (2012). There were 12 items with a maximum total score of 26. The questionnaire contained a self-report item: “What do you think of your knowledge of Chinese poetry?” ranging from 1(extremely low) to 7 (extremely high), five gap fillings (five points in total), five multiple-choice questions (10 points in total), and one subjective question (“Please write as much as you can about your understanding on the pastoral poetry”) which counted a total of four points. Two trained raters rated the prior knowledge test, and the inter-rater reliability was 0.89.

### Procedure

The participants were first informed of the procedure by the experimenter who was familiar with the procedure. Then, they completed the prior knowledge questionnaire, positive emotion questionnaire for the first time (PE1), and a demographic survey. They then learned the material under chromatic or achromatic conditions *via* a computer. Immediately after, the participants completed the positive emotion measure again (PE2) and then completed the esthetic preference judgment. This experiment lasted for ~40 min for each subject.

### Analysis

First, independent sample *t*-tests were performed to check for differences between the two conditions regarding prior knowledge (PE1). Then, we performed a 2 × 2 ANOVA with the colorful design in the learning material as a between-subject factor and emotion measures (PE1 and PE2) as the within-subject factors to test H1. Then, H2 was tested using an independent sample *t-*test with the internal colorful design of the learning material as the independent variable.

### Results

Table 1 shows the descriptive statistics of the control and dependent variables. The results of independent samples *t*-tests revealed no group differences between the two conditions regarding PE1, *t*_(78)_ = 1.43, *p* = 0.16, or in prior knowledge, *t*_(78)_ = 0.19, *p* = 0.85.

**Table 1:** Means and standard deviations of all variables for the two groups in the preliminary experiment.

**Variables**	**IC (*n* = 38) *M* (*SD*)**	**IN (*n* = 42) *M* (*SD*)**
Prior knowledge	11.05 (2.52)	10.95 (3.13)
Positive emotion (1)	5.58 (2.00)	5.15 (1.79)
Positive emotion (2)	5.64 (2.01)	5.15 (2.21)
Esthetic preference	11.53 (2.26)	10.10 (3.63)

*IN, internal neutral design; IC, internal colorful design*.

#### Positive Emotions

Regarding the effects of positive emotional design on emotions, the results revealed no significant main effects of emotional design in the learning material, *F*_(1,78)_ = 1.19, *p* = 0.28, or positive emotion measures, *F*_(1,78)_ = 0.04, *p* = 0.85, with no significant interaction effect, *F*_(1,78)_ = 0.03, *p* = 0.85.

#### Esthetic Preference

**Figure 2:**
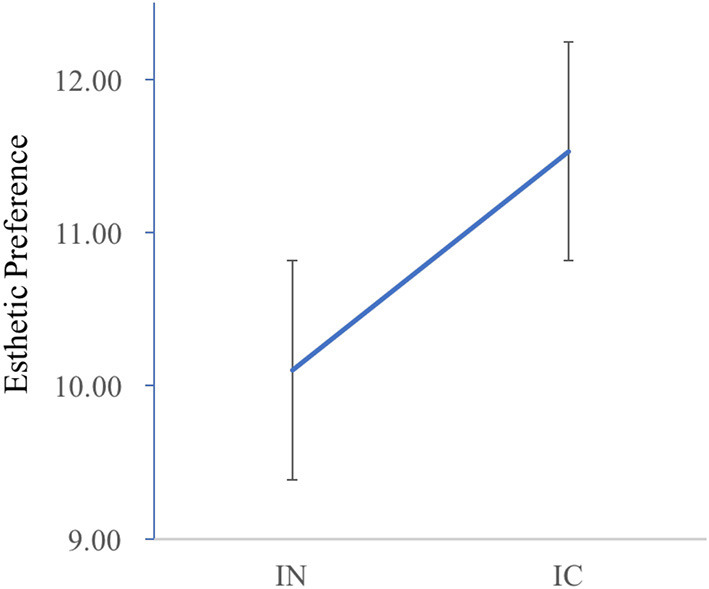
Esthetic preference in the preliminary experiment.

Regarding the effect of colorful design on esthetic preference of learners, the result revealed a significant difference, *t*_(78)_ = 2.09, *p* = 0.04, *d* = 0.47, with the colorful design inducing higher esthetic preference than the neutral design and the difference was displayed in Figure 2.

#### The Verification of the Emotional Films

##### Selection of Emotional Films

Regarding the external emotion induction videos, we employed *Mr. Bean* and *March of the Penguins* as the positive and neutral condition (Gong et al., 2017) with a total length of 346 and 330 s, respectively. The films were clips selected from the corresponding movies and edited with Photoshop.

##### Measurement and Subjects

Both clips were rated on a 7-point scale (1 = very negative, 7 = very positive) by 31 subjects (14 males, age: *M* = 14.37, *SD* = 0.67) who did not participate in multimedia learning.

##### Analysis

A paired sample *t*-test was employed to test H3.

##### Result

The paired sample *t*-test suggested that emotions induced by *Mr. Bean* were more positive than those by the *March of the Penguins* [*M* = 6.15, *SD* = 1.79; *M* = 3.95, *SD* = 1.98; *t*_(30)_ = 5.86, *p* < 0.01, *d* = 0.72].

### Brief Discussion

A preliminary experiment was conducted to verify the emotional charge of the experimental materials, such as internal colorful designed learning material and external emotion induction film clips.

The results showed that the colorful learning material did not induce more positive emotions than the neutral material, suggesting a lack of support for H1. However, compared with the neutral material, the internal colorful designed learning material induced a higher esthetic evaluation of the poem than the neutral one, which supported H2. Furthermore, emotions evoked by positive external emotion videos were more positive than those by neutral ones, supporting H3.

In terms of positive emotion, there was no significant difference between the two learning materials. That is, the internal colorful design did not induce more positive emotions than the neutral one. Many studies on emotional design have used a combination of multiple elements, such as color and anthropomorphism (Plass et al., 2014; Gong et al., 2017), and positive emotions induced in learners would increase with the increase in emotional design element amount (Uzun and Yildirim, 2018). In this study, the difference between positive and neutral conditions was only in color. This use of a single element may have weakened the emotion induction effect. This was consistent with the study of Park (Park et al., 2015), who also failed to induce positive emotions by only anthropomorphism to design learning material. Moreover, we did not induce the personification design because the poetry text already contained characters (the old and the young), thus it was not possible to use the anthropomorphic emotional design element further. Even by employing a combination of colors, shapes, and anthropomorphism, Li et al. (2020) did not successfully evoke more positive emotions, which were measured by galvanic skin response and electroencephalogram instruments objectively, in internal positive conditions than the neutral ones. In line with Li et al. (2020), the learning material in this study was presented in a system-paced manner, which meant that learners did not have control over their learning process. This may have resulted in disappointment and boredom of learners according to the control-value theory of academic emotions (Pekrun, 2006).

In terms of esthetic appreciation, the colorful designed learning material induced higher esthetic preferences in learners. Consistent with prior research and compared with achromatic learning material, the colorful material based on real objects made a more harmonious impression (Palmer et al., 2013), which may suggest that preference for colorful pictures contained in learning material may promote higher esthetic appreciation in colorful condition. In addition, the higher esthetic preference in the colorful design condition could be attributed to the age characteristics of middle school students where they were in the concrete image thinking period (Hao et al., 2019); thus, they preferred learning contents involving chromatic color, which is more vivid than the grayscale ones (Prensky, 2001; Gong et al., 2017).

Regarding the inducing effect of external induction films, *Mr. Bean* induced more positive emotions than *March of the Penguins*. Inducing emotions through movie clips is more intuitive and vivid, which can attract the attention of subjects, and is widely used to induce emotions before learning (Plass et al., 2014; Gong et al., 2017). *Mr. Bean*, which delivers a clumsy and naive performance, is obviously more emotionally contagious for middle school students than *March of the Penguins*, which is a relatively objective documentary.

Findings from the preliminary experiment suggested that the internal positive emotional design in the poem learning material can improve esthetic preference, an emotional component in esthetics that is especially relevant in poetry appreciation and is appropriate for middle school teenagers. Meanwhile, the emotion induced by the positive emotion film clip was more positive than that by the neutral one; therefore, they were assumed to be suitable for subsequent formal experiments.

## The Formal Experiment

To investigate whether the combination of external emotion induction and internal emotional design can enhance positive emotions of middle school students, esthetics, and learning performance in Chinese poetry, a formal experiment was conducted.

### Method

#### Participants and Design

When conducting the G^*^Power analysis with G^*^Power (version 3.1.9.2), we set the effect size (partial η^2^) as 0.25, α as 0.05, and power as 0.8, and the ideal total sample size was 179 with 45 in each group. Overall, 166 participants (77 males and 89 females; age: *M* = 15.05, *SD* = 0.99) with normal or corrected-to-normal vision were recruited.

We used a 2 × 2 between-subjects design with external emotion induction (positive vs. neutral) as one factor and internal emotional design (positive vs. neutral) as the other. The participants were randomly assigned to learn the material of a Chinese poem, similarly as in the preliminary experiment, under one of the four conditions:

Group 1: External positive emotion induction and internal positive emotional design condition (*n* = 44).

Group 2: External positive emotion induction and internal neutral emotional design condition (*n* = 38).

Group 3: External neutral emotion induction and internal positive emotional design condition (*n* = 41).

Group 4: Neutral under both external and internal emotional design conditions (*n* = 43).

### Materials

The experimental materials employed were the same as those utilized in the preliminary experiment.

### Measures and Procedures

The emotion measures, esthetic preference, and control measures were identical to those in the preliminary experiment. The other measures are described below.

#### Motivation

A 7-point self-report instrument containing seven items developed by Isen and Reeve (2005) was used to measure the motivation of learners (Shangguan et al., 2020b). The participants rated their motivation of the learning experience (e.g., “The learning material aroused my curiosity”) with 1 = strongly disagree to 7 = strongly agree. The final motivation score was calculated by averaging all response scores (coefficient *a* = 0.89).

#### Cognitive Load

Two items measured cognitive load of learners concerning different constructs (Deleeuw and Mayer, 2008). They were “How easy or difficult was the material to understand?” (Kalyuga et al., 2000) and “How much mental effort did you invest in studying the material?” (mental effort) (Paas, 1992). Both were 9-point rating scales and were included in the final cognitive load scores (Park et al., 2015).

#### Retention and Transfer Tests

The subjects' recognition, remembering, and reproduction of the learning material was investigated by a retention test, with a maximum score of 12, including three multiple-choice questions (For example, “What is the motif of the poem?”; six points in total) and two subjective questions [For example, “The description of ‘涧影见松竹, 潭香闻芰荷’ (The clear water reflects pine and bamboo, and the water emits the fragrance of lotus) is exquisite, try to elaborate it”; six points in total]. Answers to these questions were presented in the learning material. The retention test was rated by two trained raters with sufficient inter-rater reliability (0.9).

A transfer test was conducted to investigate the overall comprehension and appreciation of the subjects of poetry of the same motif (pastoral poetry of the Tang Dynasty). The maximum score was 17 points with three multiple-choice questions (seven points in total; for example, “Which of the following poems is not idyllic?”) and two subjective questions (10 points in total; for example, “The description of ‘荷风送香气, 竹露滴清响’ ‘The fragrance of the lotus rose far in the wind, and the dew on the bamboo leaves dropped into the water' is exquisite, try to appreciate it briefly.”) The five items were compiled regarding the representatives of pastoral poetry, writing objects, writing techniques, and thought expressions. The inter-rater reliability of the transfer test by the two trained raters was 0.86.

### Procedure

**Figure 3:**
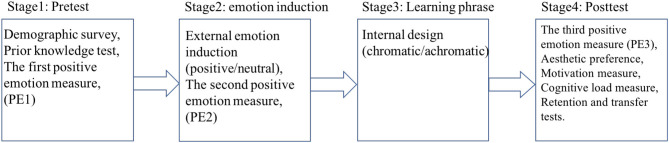
Procedure of the formal experiment.

The participants were first informed of the procedure by the experimenter who was familiar with the procedure. Then, they completed the prior knowledge questionnaire, positive emotion questionnaire for the first time (PE1), and a demographic survey. Next, the participants watched one of the two external emotion induction films and responded to the positive emotion measures (PE2) before the learning stage. Thereafter, they learned the poem material under the colorful or achromatic condition. Immediately after learning the poem, the participants completed the positive emotion measure again (PE3), and then the esthetic preference judgment, cognitive load questionnaire, motivation questionnaire, and retention and transfer tests. The complete procedure is illustrated in Figure 3.

### Analyses

First, a one-way ANOVA was performed to explore the differences between conditions of PE1 and prior knowledge, and an independent samples *t-*test was performed to check the manipulation of external emotion induction. Then, repeated measures analysis of covariance (a 2 × 4 RM-ANCOVA) was conducted with the positive emotion measures (PE2 and PE3) as the within-subject factors and the four conditions as the between-subject factors to check for manipulation. Paired sample *t*-tests were performed to examine the changes in positive emotions in the four conditions during the learning process. In addition, we performed an independent samples *t*-test to further examine the esthetic preference of Groups 3 and 4, whose external induction was the neutral film and which may have been regarded as emotionally homogeneous to the two conditions in the preliminary experiment, to examine the consistency of the esthetic emotional charge of the poem videos. Thereafter, we performed 2 × 2 ANCOVAs with internal colorful design and external emotion induction as between-subject factors; prior knowledge as covariates; and positive emotions scores, esthetic preference, motivation and cognitive load, and learning performance as dependent variables to test H4, H5, H6, and H7.

### Results

Table 2 shows the descriptive statistics of all control and dependent variables. Preliminary analyses were conducted to examine the possible differences in baseline mood (indicated by PE1) and prior knowledge. The PE1 scores were compared between the four groups [*F*_(3,162)_ = 0.42, *p* = 0.74]. The differences in prior knowledge between the four conditions were significant, *F*_(3,162)_ = 12.34, *p* < 0.001, η^2^_*p*_ = 0.19; follow-up analysis indicated that scores of Group 1 on prior knowledge were comparable with those of Group 4 (*p* = 0.99) and prior knowledge scores of Group 2 were comparable to those of Group 3 (*p* = 0.36). However, prior knowledge scores of Group 1 were significantly lower than those of Groups 2 and 3, *p* < 0.001 and *p* < 0.001, respectively; prior knowledge of Group 4 was significantly lower than those of Groups 2 and 3 (*p* < 0.001 and *p* < 0.001, respectively). Thus, prior knowledge was treated as a control variable in the following analyses. In addition, one way ANOVA revealed that the positive video (*Mr. Bean*) induced more positive emotion than the neutral video (*March of the Penguins*) before the learning of the Chinese poem, *F*_(3,162)_ = 6.93, *p* < 0.001, and further post-testing (LSD) found that the PE2 of Groups 1 and 2 were comparable (*p* = 0.71). In addition, the PE2 of Group 3 was comparable to that of Group 4 (*p* = 0.24), while that of Group 1 was significantly higher than that of Groups 3 and 4 (*p* < 0.01, *p* < 0.01). Furthermore, the PE2 of Group 2 was also significantly higher than that of Groups 3 and 4 (*p* < 0.01, *p* < 0.001).

**Table 2:** Means and standard deviations of all variables for the four groups in the formal experiment.

**Variables**	**EPIC (*n* = 44) *M* (*SD*)**	**EPIN (*n* = 38) *M* (*SD*)**	**ENIC (*n* = 41) *M* (*SD*)**	**ENIN (*n* = 43) *M* (*SD*)**
Prior knowledge	10.18 (3.11)	12.30 (2.37)	12.90 (2.37)	10.20 (2.45)
Positive emotion 1	5.57 (1.67)	5.94 (1.67)	5.61 (1.68)	5.52 (2.26)
Positive emotion 2	6.48 (1.67)	6.97 (1.75)	5.48 (1.64)	5.50 (2.34)
Positive emotion 3	5.85 (1.65)	5.93 (2.05)	5.58 (1.69)	5.57 (2.31)
Esthetic preference	10.50 (2.63)	10.92 (2.94)	10.83 (2.53)	9.21 (3.19)
Retention	6.96 (1.85)	7.16 (2.21)	6.93 (2.03)	4.74 (2.69)
Transfer	7.04 (2.69)	6.42 (2.54)	7.22 (2.26)	4.39 (2.00)
Perceived difficulty	4.68 (1.68)	5.29 (1.86)	5.22 (1.72)	5.13 (2.07)
Mental effort	5.96 (1.68)	6.24 (1.90)	6.02 (1.70)	6.09 (1.98)
Motivation	4.52 (1.66)	4.94 (1.70)	4.84 (1.57)	3.93 (1.47)

*EPIC, external positive induction and internal colorful design; EPIN, external positive induction and internal neutral design; ENIC, external neutral induction and internal colorful design; ENIN, external neutral induction and internal neutral design*.

#### Positive Emotions

First, the 2 × 4 RM-ANCOVA with the four conditions as the between-subject factors and positive emotion measures (PE2 and PE3) as repeated measures revealed a significant main effect of the conditions, *F*_(3,161)_ = 3.15, *p* = 0.03, η^2^_*p*_ = 0.06, and an insignificant main effect of positive emotion measures, *F*_(3,161)_ = 0.44, *p* = 0.51. The interaction effect was significant, *F*_(3,161)_ = 5.82, *p* < 0.001, η^2^_*p*_ = 0.1. Concerning whether the positive emotion (from PE2 to PE3) changed significantly during the learning process, the results of paired sample *t*-tests indicated that for Group 1, the scores on positive emotion reduced significantly from PE2 to PE3, *t*_(43)_ = 3.02, *p* < 0.01, *d* = 0.68; for Group 2, positive emotions decreased significantly from PE2 to PE3, *t*_(37)_ = 4.7, *p* < 0.001, *d* = 0.76; and for Groups 3 and 4, the positive emotions remained comparable from PE2 to PE3, *t*_(40)_ = −0.7, *p* = 0.49; *t*_(42)_ = −0.37, *p* = 0.72.

Regarding the effects of external emotion induction and internal colorful design on positive emotions (PE3), the results of the 2 × 2 ANCOVA showed no main effect of external emotion induction, *F*_(1,161)_ = 1.2, *p* = 0.28, or a main effect of internal colorful design, *F*_(1,161)_ = 0.3, *p* = 0.86. Furthermore, no interaction effect was found, *F*_(1,161)_ = 0.05, *p* = 0.83.

**Figure 4:**
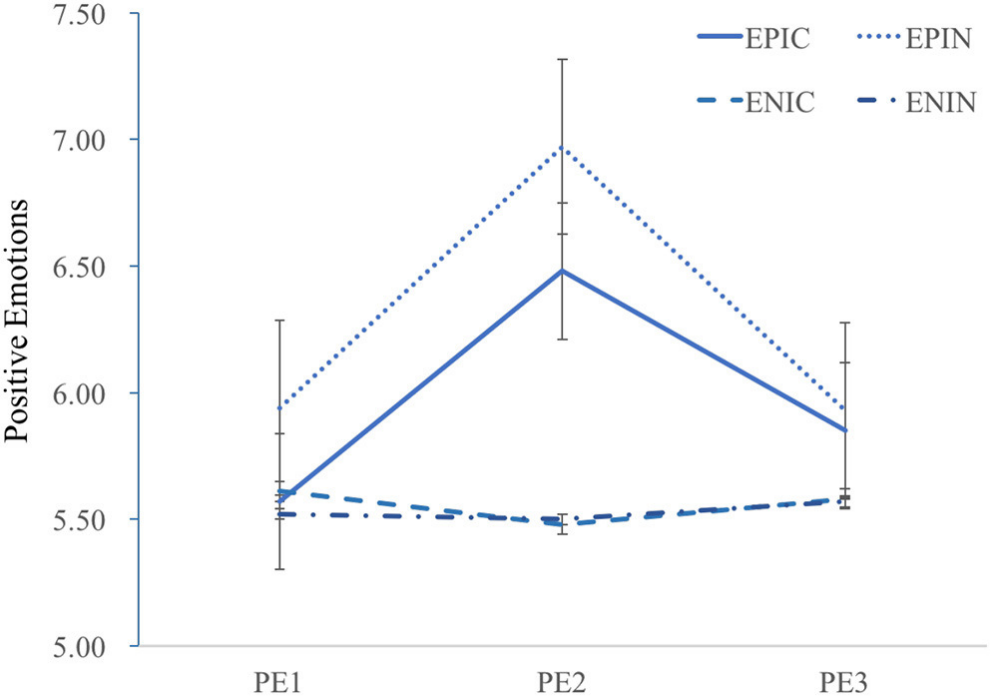
Positive emotions (PE1, PE2, and PE3) in the formal experiment.

The results of PE1, PE2, and PE3 are presented in Figure 4 to enable readers to have an intuitive understanding of emotional changes during the experiment.

#### Esthetic Preference

Concerning esthetic preference of learners as a dependent measure, the results of 2 × 2 ANCOVAs showed no significant main effects of external emotion induction or internal colorful design, *F*_(1,161)_ = 2.68, *p* = 0.1; *F*_(1,161)_ = 1.68, *p* = 0.2. Furthermore, no interaction effect was observed, *F*_(1,161)_ = 2.46, *p* = 0.12. However, an interesting and consistent result was revealed by the independent *t*-test, where the esthetic preference of Group 3 was significantly higher than that of Group 4, *t*_(82)_ = 2.57, *p* < 0.012, *d* = 0.56.

#### Cognitive and Motivation Outcomes

The effects of external emotion induction and internal colorful design on learning outcomes were examined by 2 × 2 ANCOVAs, with prior knowledge as a control variable and retention and the transfer test scores as dependent measures.

The main effects of external emotion induction and internal colorful design on retention were both significant, *F*_(1,161)_ = 12.45, *p* < 0.01, η^2^_*p*_ = 0.07; *F*_(1,161)_ = 7.05, *p* < 0.01, η^2^_*p*_ = 0.04. The retention scores under the external positive emotion induction condition were significantly higher than those under the external neutral emotion induction condition. Performance on retention showed the same pattern as the internal colorful design. Furthermore, the interaction between the two factors was not significant, *F*_(1,161)_ = 2.72, *p* = 0.1.

Significant main effects of external emotion induction and internal colorful design on transfer scores were also revealed, *F*_(1,161)_ = 5.62, *p* = 0.02, η^2^_*p*_ = 0.03; *F*_(1,161)_ = 17.53, *p* < 0.001, η^2^_*p*_ = 0.1. The transfer scores for positive external emotion induction conditions were significantly higher than those for neutral external conditions. Performance on transfer tests suggested the same tendency as the internal colorful design. The interaction between the above factors did not significantly affect the transfer performance, *F*_(1,161)_ = 0.33, *p* = 0.86.

**Figure 5:**
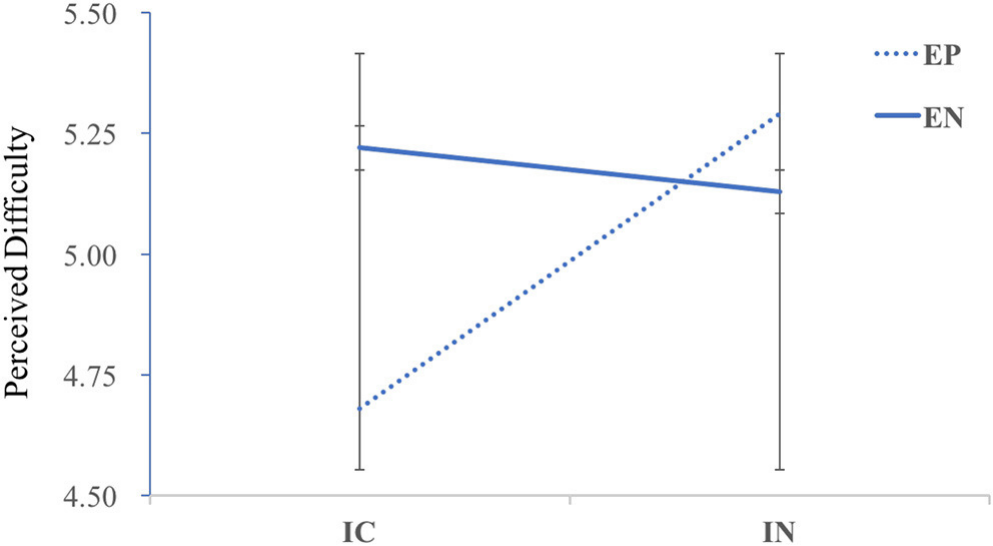
Perceived difficulty by condition in the formal experiment.

Concerning perceived difficulty, mental effort, and motivation of learners as dependent measures, and prior knowledge as control variables, the results of the 2 × 2 ANCOVA showed no significant main effects, *Fs* < 0.76, *p* > 0.05. However, the interaction on perceived difficulty was significant, *F*_(1,161)_ = 9.55, *p* < 0.05, η^2^_*p*_ = 0.06, and LSD-corrected *post-hoc* tests revealed that when positive emotion was induced before learning, the perceived difficulty of internal colorful design was significantly lower than the internal neutral condition, *F*_(1,161)_ = 4.62, *p* = 0.03, η^2^_*p*_ = 0.03. While the neutral video was presented before learning, perceived difficulty showed a reversal pattern where the perceived difficulty was significantly lower in the achromatic condition than in the chromatic condition, *F*_(1,161)_ = 5.9, *p* = 0.02, η^2^_*p*_ = 0.04 (see in Figure 5). No other interaction effects were revealed, *Fs* < 0.57, *p* > 0.05.

### Brief Discussion

The formal experiment examined whether the combination of external emotion induction and internal colorful design would promote the appreciation and learning of Chinese poems. The results showed that external emotion induction significantly improved positive emotions of learners when they entered the learning situation, thus, partially supporting H4. The combination of external emotion induction and internal colorful design did not improve the esthetic evaluation of poems; however, the color learning material boosted appreciation of learners of the poem compared with the gray scale one when the external film clip was neutral, therefore, partially supporting H5. Moreover, the combination reduced perceived difficulty (partially supporting H6) and enhanced retention and transfer performance of learners (supporting H7).

Regarding positive emotions, consistent with previous research (Plass et al., 2014; Gong et al., 2017), the external positive emotional video successfully induced more positive emotions in learners than the neutral one (indicated by PE2). According to the CATLM (Moreno, 2006; Moreno and Mayer, 2007), the positive emotions induced by external manipulation may influence the learning process by activating the topic-related prior knowledge of a learner and driving learners to recruit cognitive resources in subsequent similar situations, thus, promoting comprehension of learners and retention of the poem, and boosting their transfer performance. Furthermore, positive emotion fosters more holistic processing of information, which coincides with more creative thinking (Isen, 1999; Bless and Fiedler, 2006; Knörzer et al., 2016; Beege et al., 2018) and, therefore, may promote deeper learning of Chinese poetry, which is also a creative process.

Concerning esthetic preference judgment, no main effect or interaction effect of external emotion induction and internal colorful design was found. Compared with the preliminary experiment in which internal colorful design promoted esthetic preference of learners for the poem using a chromatic design element, the formal experiment introduced external emotion induction by employing emotional films, with *Mr. Bean* being used in the external positive condition. The clumsy and naive performance of *Mr. Bean* successfully evoked more positive emotions. Although poetry always possesses an emotional component that facilitates esthetic preference in readers, the emotion of poetry is usually captured through referential linguistic conventions, such as metaphors and imagery (Piirto, 2011). This may be less emotionally contagious for middle school students than *Mr. Bean*, which has been famous for its amusement for over 30 years worldwide. The positive emotions evoked by external induction videos may mask emotional involvement of the poem perceived by learners, thus, resulting in a null effect on esthetic preference judgment. Moreover, esthetic preference is primarily driven by high affective arousal (Kraxenberger and Menninghaus, 2017), and the arousal of *Mr. Bean* was much higher than the poem containing neutral key emotional tonality; thus, the film showed higher esthetic preference. However, it should not be ignored that when the external emotion induction is neutral (Groups 3 and 4), which we may regard as emotionally homogeneous to the preliminary experiment, the internal colorful design made the poem more appealing to learners. This was consistent with the preliminary experiment, indicating that the internal colorful design successfully induced emotional esthetics of teenagers in poetry appreciation.

Regarding the cognitive load outcomes, it was found that when the external video successfully induced positive emotions, perception difficulty of the learners was significantly lower in the internal colorful design condition than in the internal neutral condition; when the external induced video was neutral, the perception difficulty of the internal neutral condition was significantly lower than that of the internal colorful condition. That is, the congruency of external and internal emotions reduces perceived difficulty. The mood-affect congruency (Kim and Pekrun, 2014; Beege et al., 2018; Schneider et al., 2019) facilitated the access and retrieval of topic-related experiences through stronger activation, thus, reducing perceived difficulty. Concerning motivation, no effects of external emotion induction or internal colorful design were revealed, which was in line with previous research (Kumar et al., 2016; Navratil et al., 2018), suggesting that the learning material was designed in a way that promotes positive emotions rather than motivation (Knörzer et al., 2016; Stark et al., 2018; Shangguan et al., 2020a). More explorations need to be implemented to motivate teenagers in multimedia learning.

## General Discussion

This study investigated the influence of emotional designs on Chinese poetry esthetics and learning in multimedia learning. The results showed that the use of color in the internal colorful design in the learning material of Chinese poetry did not induce more positive emotions than the neutral one (lack of support for H1) and could significantly improve esthetic preference of learners (in support of H2). The external positive films evoked more positive emotions than the neutral film (in support of H3) in the preliminary experiment. In the formal experiment, positive external emotion induction improved positive emotions when entering instructional situations (in partial support of H4). When the external induction was neutral, the colorfully designed learning material achieved a higher appreciation of the poem in learners than the gray scale one (partially supporting H5). Mood (induced by external induction)-affect (internal colorful design) congruency reduced perceived difficulty of learners (in partial support of H6), and the combination of external emotion induction and internal colorful design boosted retention and transfer performance (supporting H7). Overall, the findings partly replicated the results in natural science disciplines and supported the CATLM theory. This study extended the discipline fields of existing emotional designs in multimedia learning to humanities by examining the effects of emotional designs on learning and appreciation, which may reflect the unique disciplinary attributes of Chinese poetry.

Regarding the positive emotion outcomes, the single internal colorful design element did not successfully evoke positive emotions (Park et al., 2015), and the external emotion induction induced short-lived positive emotions (Gong et al., 2017). Consistent with previous literature, these results suggest that the means of inducing emotions have inherent characteristics; one-dimensional manipulation of internal colorful design may have limited effects in accumulating enough positive emotions captured by scales, while the external mood induction effect was unsustainable throughout the learning process. Further research should be conducted to explore more design variations, such as design elements at the behavioral level implied in the control-value theory of academic emotions (Pekrun, 2006), to evoke positive emotions effectively and efficiently. Moreover, esthetic preference of learners, an emotional indicator in poetry appreciation, for the poem was higher in the positive condition than in the neutral one in the preliminary experiment, which may indirectly verify the effectiveness of the internal colorful design.

Regarding cognitive outcomes, mood-affect congruency reduced perceived difficulty. The effect of mood-affect congruency on cognitive load could be explained by an associative network (Kim and Pekrun, 2014) where emotions are inextricably linked to events in daily life; hence, objective knowledge is stored emotionally in memories. Mood-related knowledge would be more easily available if the mood is experienced before retrial (Levine and Pizarro, 2004), following which the combination of positive external emotion induction and internal colorful design would decrease perceived difficulty and is conducive to deeper processing of the poem. Furthermore, learning outcomes in positive conditions were better than those in neutral conditions. This is not surprising as emotion always interacts with other cognitive processes (Knörzer et al., 2016) and is a crucial factor that influences learning of individuals in educational settings (Pekrun, 2006). It also affects creativity (Hu and Wang, 2010). Positive emotions are associated with a wider attention span, global information processing (Fredrickson, 2004), and improved creativity implicated in the appreciation of poetry, which may explain the better learning performance in positive conditions regardless of internal colorful design or external emotion induction.

Although science, committed to revealing the truth of nature, and art (humanities), committed to create beauty and to express inner desires and emotions, are two different aspects of human activities and inquiries, the findings replicated the results of multimedia learning in natural science disciplines. This supports the statement, “In education, the perfect combination of science and humanities is the hope of cultivating talents who can meet the development needs of the new century” made by Li Zhengdao, a Chinese physicist.

### Implications and Limitations

Two theoretical implications can be derived from this study. First, according to the purposes of prior studies, results from emotional design studies on natural sciences, such as biology (Park et al., 2015; Knörzer et al., 2016), physics (Mayer, 2005; Gong et al., 2017; Uzun and Yildirim, 2018), and astronautics (Kühl et al., 2018) can be transferred to humanities. Meanwhile, humanities, such as story reading (Takacs and Bus, 2016) and appreciation of poetry, seems to require learners to actively and creatively construct and have a closer bond with the relevant knowledge and experience of recipients. This suggests that the measures of learning effect may not be limited to conventional measures of retention, comprehension, and transfer. Therefore, it is necessary to seek other indicators that fit the attributes of the subject, such as esthetic evaluations. In practice, when implementing emotional designs in multimedia learning, not only is the emotional design of the learning material necessary but also the influence of existing emotions of learners when entering educational situations on subsequent learning should be considered.

Some limitations that should be considered: first, the topics of poems ranged throughout inspiration by love and desire, nature, social injustice, dreams, and many other situations (Piirto, 2011). Only one pastoral poem was studied in this research; thus, whether the results could be extended to other themes remain unanswered. In addition, the number of participants in different conditions in the preliminary and formal experiments was not the same, which may have resulted in experimental deviations. Future research should pay more attention to the choice of the number of participants to avoid possible errors. Moreover, as for the measurement of emotion, we used self-reported items before and after learning instead of an objective, continuous measurement. Finally, demographic variables, such as gender (Castroalonso et al., 2019) and age (Shangguan et al., 2020a), which may also have impact multimedia learning, need to be further investigated.

## Data Availability Statement

The original contributions presented in the study are included in the article/supplementary material, further inquiries can be directed to the corresponding author/s.

## Ethics Statement

The studies involving human participants were reviewed and approved by Ethical Committee of the School of Psychology at Central China Normal University. Written informed consent to participate in this study was provided by the participants' legal guardian/next of kin. Written informed consent was obtained from the individual(s), and minor(s)' legal guardian/next of kin, for the publication of any potentially identifiable images or data included in this article.

## Author Contributions

YW: conceptualization, acquisition, collection, analysis, interpretation, and drafting. ZZ: conceptualization, supervision, and validation. SG: conceptualization, interpretation, revision of the study, and validation. DJ: revision of the draft. JL: design of the learning materials and tests. All authors contributed to the article and approved the submitted version.

## Conflict of Interest

The authors declare that the research was conducted in the absence of any commercial or financial relationships that could be construed as a potential conflict of interest. The reviewer JY declared a shared affiliation with several of the authors, YW, ZZ, SG, and DJ, to the handling Editor at time of review.

## Publisher's Note

All claims expressed in this article are solely those of the authors and do not necessarily represent those of their affiliated organizations, or those of the publisher, the editors and the reviewers. Any product that may be evaluated in this article, or claim that may be made by its manufacturer, is not guaranteed or endorsed by the publisher.
